# Anisotropic shock responses of nanoporous Al by molecular dynamics simulations

**DOI:** 10.1371/journal.pone.0247172

**Published:** 2021-03-17

**Authors:** Xia Tian, Kaipeng Ma, Guangyu Ji, Junzhi Cui, Yi Liao, Meizhen Xiang

**Affiliations:** 1 College of Mechanics and Materials, HoHai University, Nanjing, China; 2 College of Civil and Transportation Engineering, HoHai University, Nanjing, China; 3 LSEC, ICMSEC, Academy of Mathematics and Systems Science, CAS, Beijing, China; 4 School of Mathematical Sciences, University of Chinese Academy of Sciences, Beijing, China; 5 School of Mechanical Engineering, Southwest Petroleum University, Chengdu, China; 6 Laboratory of Computational Physics, Institute of Applied Physics and Computational Mathematics, Beijing, China; University of Akron, UNITED STATES

## Abstract

Mechanical responses of nanoporous aluminum samples under shock in different crystallographic orientations (<100>, <111>, <110>, <112> and <130>) are investigated by molecular dynamics simulations. The shape evolution of void during collapse is found to have no relationship with the shock orientation. Void collapse rate and dislocation activities at the void surface are found to strongly dependent on the shock orientation. For a relatively weaker shock, void collapses fastest when shocked along the <100> orientation; while for a relatively stronger shock, void collapses fastest in the <110> orientation. The dislocation nucleation position is strongly depended on the impacting crystallographic orientation. A theory based on resolved shear stress is used to explain which slip planes the earliest-appearing dislocations prefer to nucleate on under different shock orientations.

## Introduction

Nanoporous metals play an important role in the fields of military, aeronautical engineering, energy, catalysis, environmental protection and biomedicine [1]. Many researchers have investigated the dynamics response of porous materials by experiments [2–7], theoretical methods [8, 9, 11] and numerical simulations [12–15]. For example, Levy [2] investigated the collision of a planar shock wave with a rigid porous material in a 75 mm × 75 mm shock tube. The experimental study indicated that unlike the reflection from a flexible porous material where the transmitted compression waves do not converge to a sharp shock wave, in the case of a rigid porous material, the transmitted compression waves do converge to a sharp shock wave; Kazemi-Kamyab [3] studied the interaction of moving shock waves with short length elastic porous aluminum samples of various porosities in a shock tube facility in a setup where the specimens were placed in front of a long rod of a modified Hopkinson Bar. High frequency response miniature pressure transducers and semiconductor strain gages were used to measure the pore gas pressure and the transmitted stress wave to the rod respectively; Feldgun [8] proposed a model of two-phase porous medium to simulation of dynamic behavior of metal foams. The proposed approach is demonstrated by comparison with experimental data; Following the constitutive framework developed by Molinari and Ravichandran [10] for the analysis of steady shock waves in dense metals, Czarnota et al. [11] proposed an analytical approach of steady state propagation of plastic shocks in porous metals. The results obtained in their work provided a new insight in the fundamental understanding of shock waves in porous media; Xiang et al. [12] presented systematic investigations examining the shock responses of nanoporous aluminum by nonequilibrium molecular dynamics simulations, they proposed a continuum wave reflection theory and a resolved shear stress model to explain the distribution of dislocation nucleation sites; Liao et al. [13] presented systematic investigations on energy dissipation and void collapse in graded nanoporous nickel by non-equilibrium molecular dynamics simulations. It is found that void size gradient influences the time history path of the energy dissipation; Li et al. [14] investigated shock response of nanoporous Mg by nonequilibrium MD simulations. The shock Hugoniot curves of Mg, shock void collapse mechanisms, thermodynamics characteristics and spall damage are considered in their work; Guan et al. [15] used Molecular dynamics method to investigate the dynamic response of void-included aluminum under three loading patterns, which are constant strain rate, constant-stress Hugoniostat and direct shock loadings. The simulations show a very weak dependence of the dynamic response on the loading patterns under weak loadings, where appears a similar dislocation distribution originated from the initial void.

Although there have been some studies on the shock response of nanoporous materials, discussions of the anisotropic shock responses of nanoporous materials remain unclear. Because these results can serve as important references for constructing micromechanism-based continuum models for void collapse at macro- or meso- scales, it is necessary for us to investigate how crystallographic orientations affect the plastic deformation mechanism, including the void collapse mechanism and dislocation nucleation mechanism of nanoporous materials. In the present work, we focus on investigating how crystallographic orientations affect the shock response of nanoporous Al samples under shock compression. Though quantitative comparisons between MD simulations and experiments may be inappropriate due to differences in length and time scales, MD simulations can serve as important complements to experiments to probe qualitative trends as well as underlying micro-mechanisms of material deformation and failure [11]. For shock problems, MD simulation technique allowed us to explicitly reveal not only spalling damage accumulation processes (nucleation, growth and coalescence of micro-voids) but also dynamic evolutions of local stress and temperature histories which are currently unavailable in shock experiments. The work is organized as follows, the model and methodology are addressed in Section 2, void collapse mechanisms and dislocation nucleation mechanisms are discussed in Section 3, and the work is summarized in Section 4.

## Materials and methods

**Fig 1:**
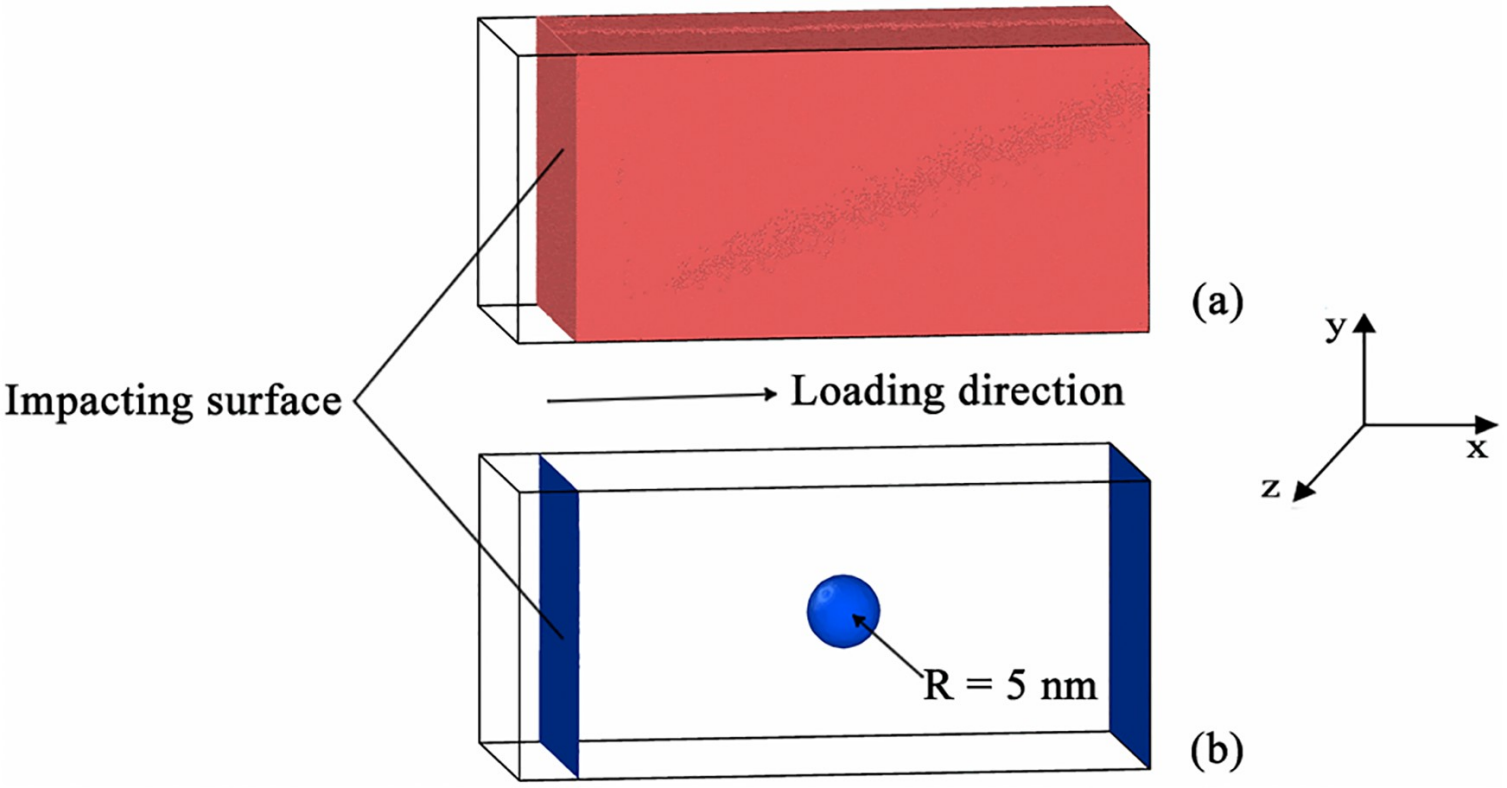
The simulation sample with a size of 80 nm × 40 nm × 40 nm. (a) The outside view of the simulated nanoporous Al sample, (b) illustration of the void located in the center of the simulated sample, the blue structures represent surfaces.

The size of nanoporous Al sample is 80 nm × 40 nm× 40 nm, and a void with radius R = 5 nm is located in the center of the sample, as shown in Fig 1. An empirical Embedded Atom Method (EAM) potential developed by Winey et al. [16] is chosen to describe the interactions between Al atoms. This EAM potential has been widely used and its accuracy under shock conditions has been well confirmed by Liao [17]. To investigate the anisotropic shock responses of nanoporous Al, five crystallographic orientations are considered in our simulations. Specifically, the *x*, *y* and *z* axes of the nanoporous Al samples are along: (1) [100], [10] and [1] crystallographic orientations; (2) [111], $\left[11\bar{2}\right]$ and $\left[\bar{1}10\right]$ crystallographic orientations; (3) [110], $\left[\bar{1}10\right]$ and [1] crystallographic orientations; (4) [112], $\left[\bar{1}10\right]$ and $\left[11\bar{1}\right]$ crystallographic orientations; (5) $\left[1\bar{3}0\right]$, [310] and [1] crystallographic orientations, respectively. The five nanoporous samples can be denoted as <100>, <111>, <110>, <112> and <130> nanoporous Al (np-Al) samples in short.

Before shock loading, the energy of the np-Al samples is minimized by conjugate gradient method. Then all the simulated np-Al samples are relaxed in the isobaric-isothermal ensemble at 300 K and 0 GPa for 50 ps to let the initial residual stress of the system be negligible comparing to the shock intensity. During the relaxation process, the residual stress decreases from about 3.9 GPa to 0.002 GPa. As the shock intensities in our simulations are larger than 3.0 GPa, the residual stress in the relaxed system is negligible comparing to the shock intensities. The shock waves in the np-Al target samples are generated by moving a rigid Al piston in the *x* direction with a fixed velocity. To inspect the void collapse and dislocation emission mechanisms under different impacting intensities, we chose three impacting velocities, a relatively low impacting velocity *v* = 0.2 km/s, a medium impacting velocity *v* = 0.5 km/s (actually, *v* = 0.6 km/s is also suitable) and a relatively high impacting velocity *v* = 1.0 km/s. Free boundary conditions are utilized along the shock loading direction (*x* direction) while periodic boundary conditions are used in *y* and *z* directions. The timestep in the relaxation and equilibrium processes is chosen to be 1 fs, and the timestep in the NEMD simulations is chosen to be 0.2 fs. MD simulations in the present work are performed by Large-scale Atomic/Molecular Massively Parallel Simulator (LAMMPS) [18], and the atomic configurations are visualized by the visualization software OVITO [19–22]. In the following discussions, dislocations are identified by the dislocation extraction algorithm (DXA), which has been implemented into OVITO as a standard modifier [22].

## Results and discussion

### Void collapse mechanisms

The overall strain is relevant to the impacting velocity and varies with time. The target is in the tightest compression state once the wave front achieves the free surface, resulting in a largest overall strain during shock loading. The maximum values of the overall strain in different lattice orientations and under distinct impacting intensities are listed in Table 1, where, *v* is the impacting velocity, *Ԑ*_*max*_ is the maximum overall strain at the moment when the wave front arrives at the free surface. In Table 1, it is found that for a certain lattice orientation, the maximum overall strain increases with the augment of the impacting intensities.

**Table 1:** Maximum overall strain at different lattice orientations and impacting velocities.

Lattice orientation	*v*	*Ԑ*_*max*_
<100>	0.2	3.40%
	0.5	7.10%
	1.0	13.10%
<111>	0.2	3.30%
	0.5	6.90%
	1.0	11.10%
<110>	0.2	3.40%
	0.5	7.10%
	1.0	11.50%
<112>	0.2	3.40%
	0.5	7.00%
	1.0	11.50%
<130>	0.2	3.40%
	0.5	7.00%
	1.0	12.50%

The unit for velocity is km/s.

**Fig 2:**
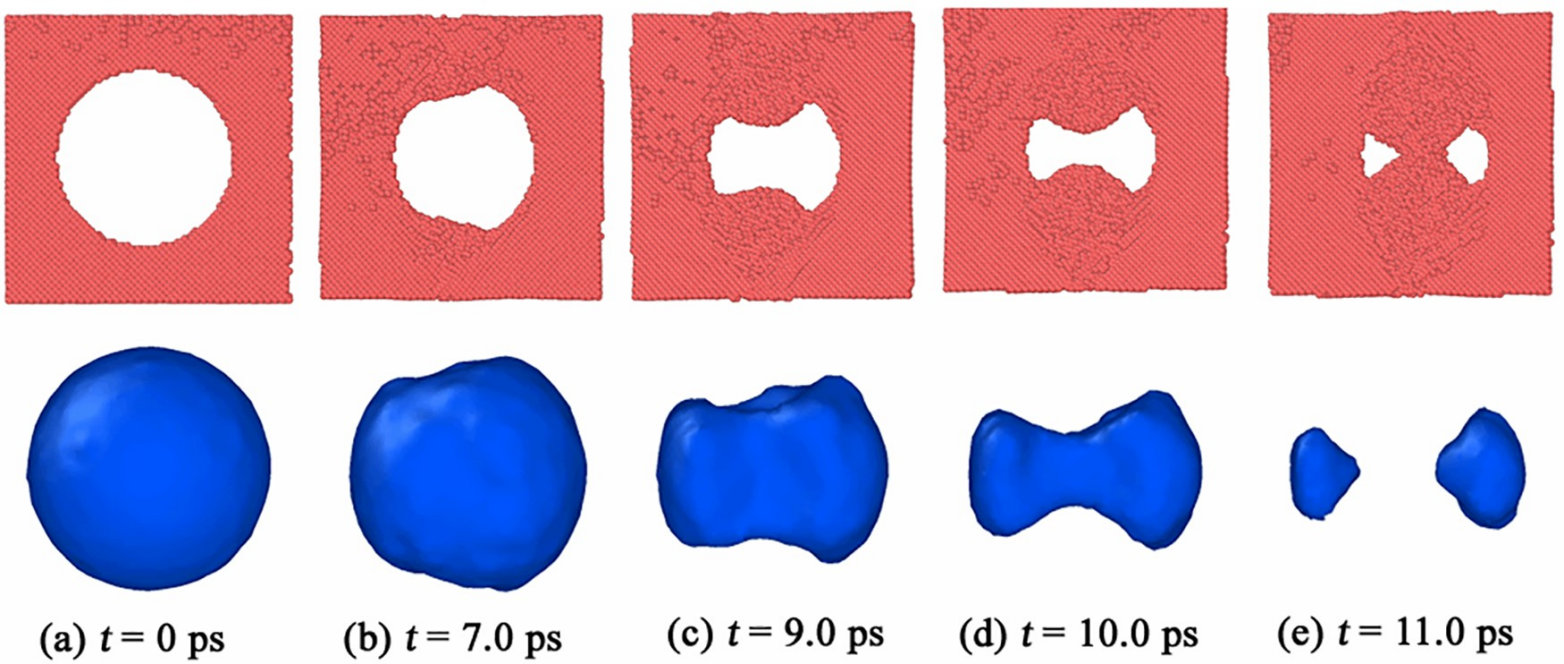
The shape evolution of void during collapse in <100> np-Al target under *v* = 0.5 km/s.

In order to intuitively describe void collapse process and accurately reveal the microscopic mechanisms, we display the void shape evolution during void collapse in <100> np-Al sample under *v* = 0.5 km/s in Fig 2. The partially collapsed void looks like a non-symmetrical dumbbell, as seen in Fig 2(a)–2(d). The original void is divided into two sub-voids at *t* = 11.0 ps, as shown in Fig 2(e). After that, the two sub-voids shrink gradually and disappear finally.

**Fig 3:**
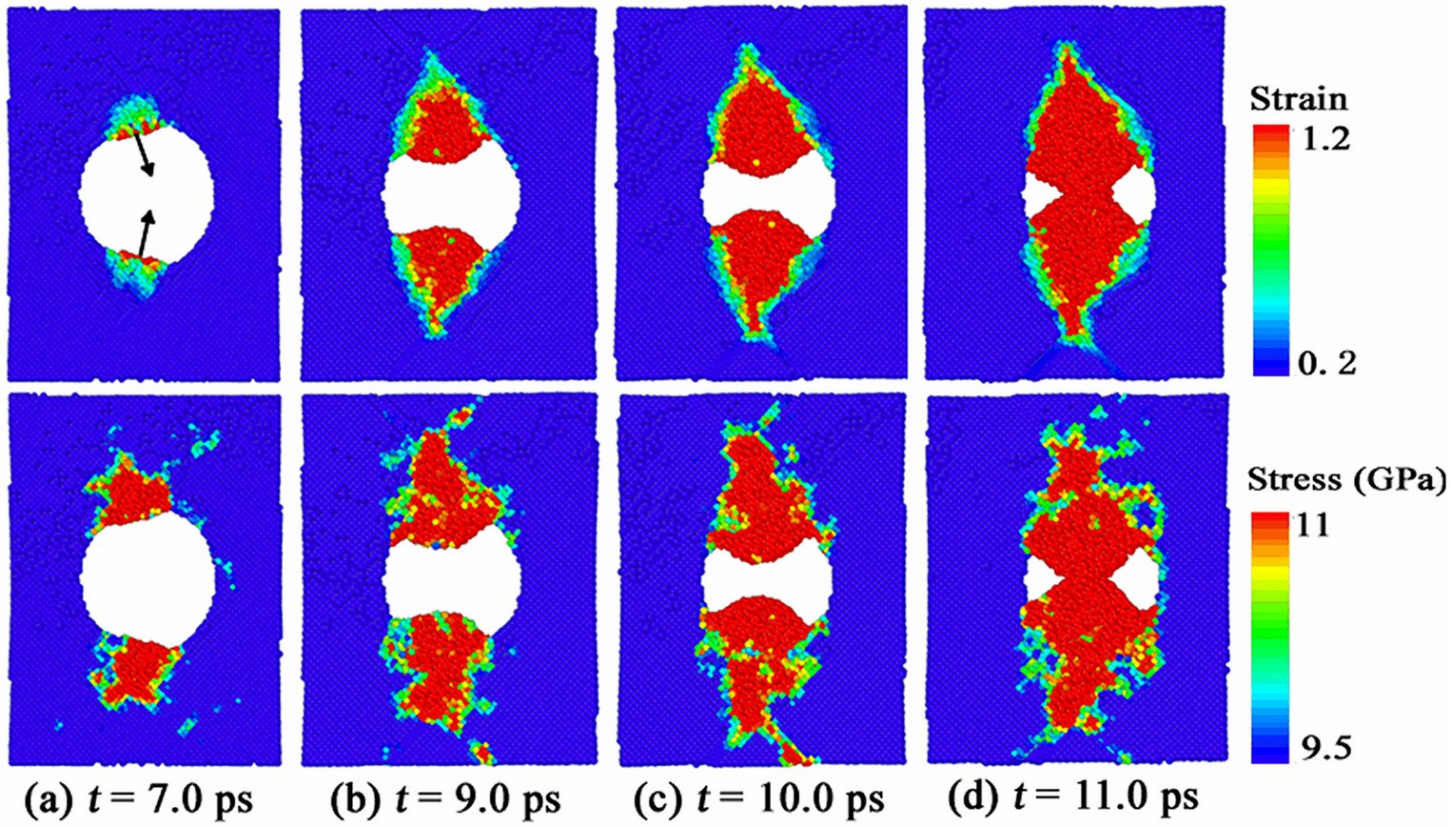
Distributions of (a) von Mises local strain and (b) von Mises stress around the collapsed void in <100> np-Al sample under *v* = 0.5 km/s.

To illustrate why the shape evolution of void during collapse happens in Fig 2, we calculated the von Mises local strain invariant around the collapsed void. The von Mises local strain invariant is calculated by “atom strain”, which is a standard modifier implemented in the open visualization tool OVITO [23, 24]. The top figures of Fig 3 display the von Mises local strain around the collapsed void in <100> np-Al sample under *v* = 0.5 km/s. From Fig 3(a), we can see that the local strain reaches the maximum value in two local regions of the upper and lower positions of the void, which leads to the shrinkage of the void to its inner space, as indicated by the black arrows in Fig 3(a); then the fields with high shear strain expand, resulting in a transverse “necking” of the void, as shown in Fig 3(b)–3(d). To discuss the collapse process of the void in depth, we also calculated the stress distributions around the void. The von Mises stress can be expressed by the following equation,

$$\sigma_{von-Mises}=\sqrt{[{\left(\sigma_{x}-\sigma_{x}\right)}^{2}+{\left(\sigma_{y}-\sigma_{z}\right)}^{2}+{\left(\sigma_{x}-\sigma_{z}\right)}^{2}+6(\tau_{{}_{xy}}^{2}+\tau_{yz}^{2}+\tau_{{}_{xz}}^{2})]/2}.$$

The von Mises stress distributions around the void under *v* = 0.5 km/s are displayed in the bottom figures of Fig 3. In Fig 3(a), we can see that the local von Mises strain reaches the maximum value in the position indicated by the black arrows, resulting in a pronounced nonequilibrium phenomenon with the neighborhood. At the same time, plastic behaviors make the local stress rise, leading to a similar distribution of the local stress with that of the strain. Similar stress and strain distributions can be found in Fig 3(b)–3(d).

**Fig 4:**
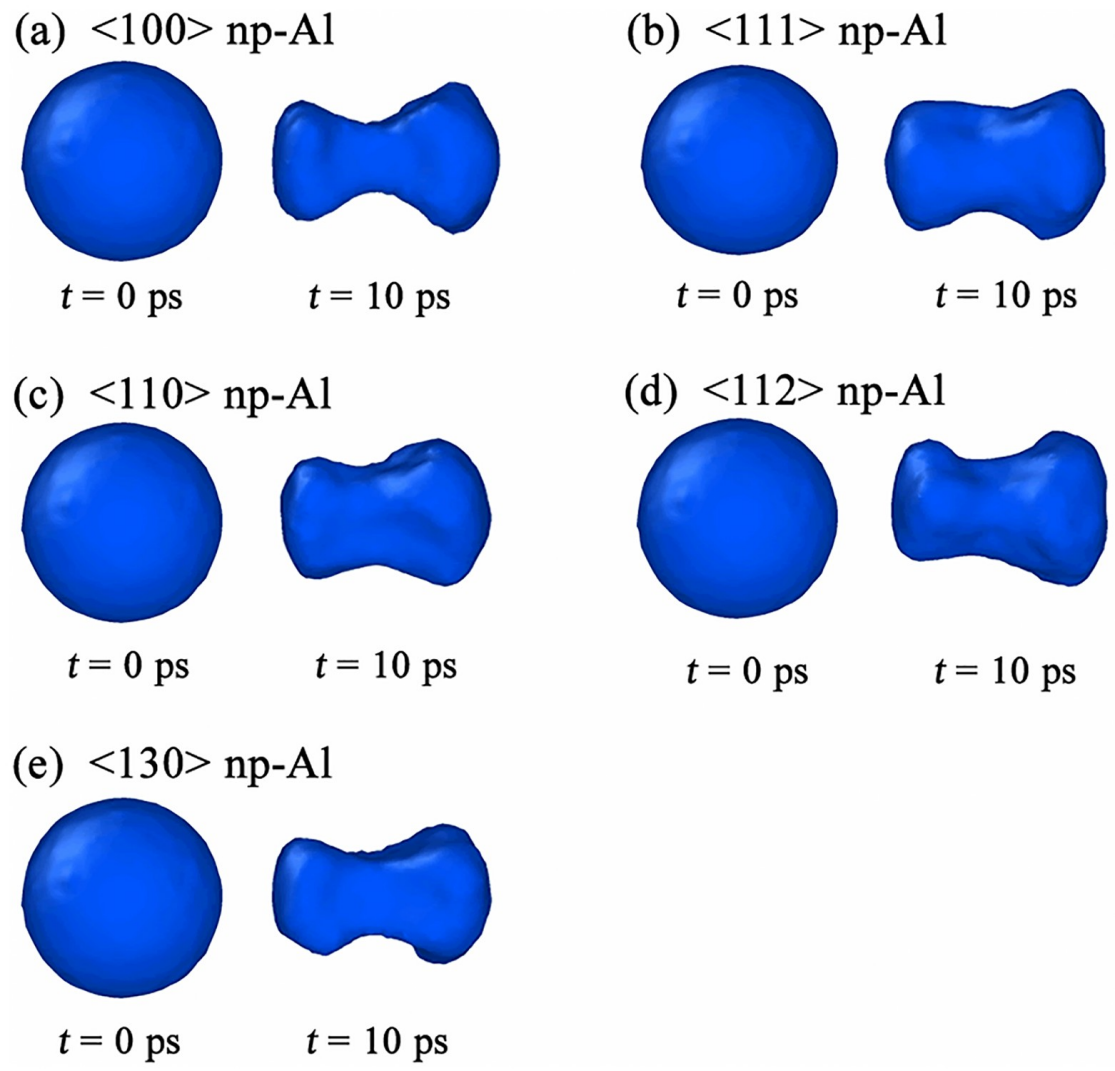
The shape evolution of void during collapse in all simulated samples under *v* = 0.5 km/s.

In our simulations, we find that the shape evolution of void during collapse has no relationship with the crystallographic orientations along shock direction. Fig 4 displays the shape of partially collapsed void in <100>, <111>, <110>, <112> and <130> np-Al targets, respectively. All collapsed voids look like non-symmetrical dumbbells, which is responsible for the strain intensity in the upper and lower positions of the void, as illustrated in Fig 3. In simulations, it is found that void collapse happens at about *t* = 11.8 ps, 12.4 ps, 12.6 ps, 12.2 ps and 12.0 ps in <100>, <111>, <110>, <112> and <130> np-Al samples, respectively. Therefore, np-Al in crystallographic orientation of <100> exhibited fastest void collapse rate under the shock of *v* = 0.5 km/s.

**Fig 5:**
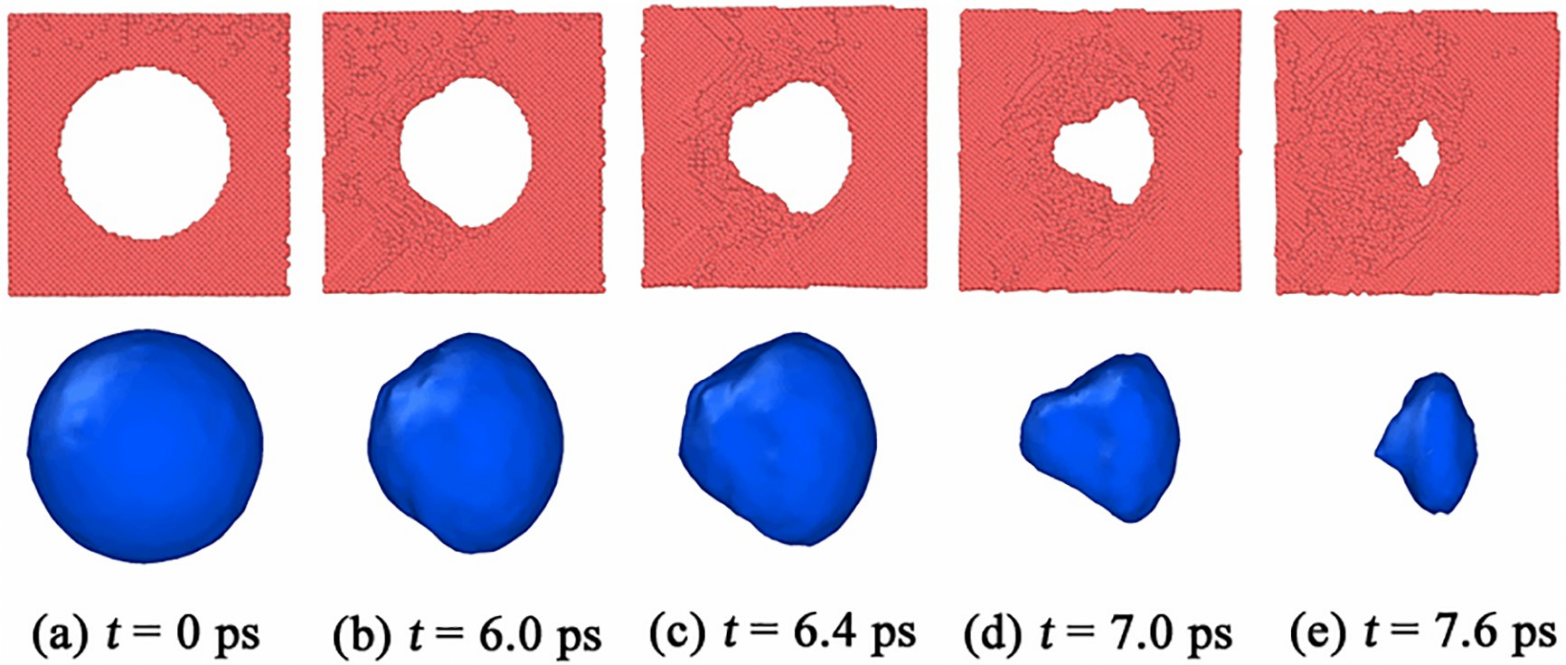
The shape evolution of void during collapse in the <100> target under *v* = 1.0 km/s.

**Fig 6:**
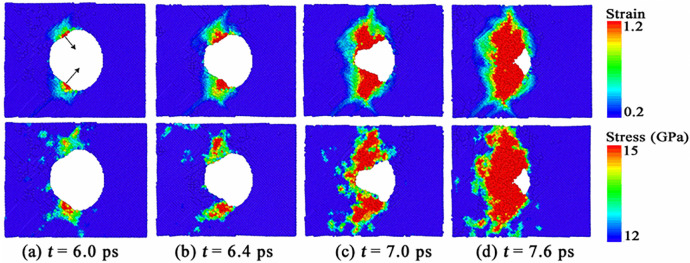
Distributions of the von Mises local strain invariant around the collapsed void in <100> np-Al target under *v* = 1.0 km/s.

Fig 5 displays the shape evolution of the void during collapse under *v* = 1.0 km/s. The shape of the partially collapsed void is like a cone, and the void shrinks along the direction that is in a certain angle with the shock direction, as shown in Fig 5(a)–5(e). Like the case of *v* = 0.5 km/s, the shape evolution of void during collapse has no special relationship with the crystallographic orientations along shock direction in the case of *v* = 1.0 km/s. Moreover, void collapse occurs at *t* = 7.8 ps, 7.2 ps, 6.8 ps, 7.0 ps and 7.5 ps in <100>, <111>, <110>, <112> and <130> np-Al samples, respectively. Thus, np-Al in crystallographic orientation of <110> collapsed fastest under the shock of *v* = 1.0 km/s. Fig 6 illustrates the von Mises local strain and von Mises stress around the collapsed void in <100> np-Al sample under *v* = 1.0 km/s. The local strain of the regions indicated by the black arrows around the void are very large, as shown in the top figure of Fig 6(a). In other words, the deformation is severe in these regions. Meanwhile, plastic behaviors make the local stress increase, resulting in a similar distribution of local stress to that of the local strain, as seen in the bottom figure of Fig 6(a). Similar stress and strain distributions can be found in Fig 6(b)–6(d). With further deformation, the high shear strain area extends to the front surface of the void, resulting in a conical void, as shown in Fig 6(b)–6(d). Unlike the plastic mechanism under *v* = 0.5 km/s, the void collapse in this case is dominated by the internal jetting mechanism [12], which leads to filling of the void vacuum in the longitudinal direction.

### Dislocation nucleation mechanisms

In this section, we focus on investigating the dislocation nucleation mechanism of np-Al at different lattice orientations under impacting intensities of *v* = 0.2, 0.5 and 1.0 km/s. Firstly, we intend to discuss which {111} slip planes the earliest-appearing dislocations prefer to nucleate on.

**Fig 7:**
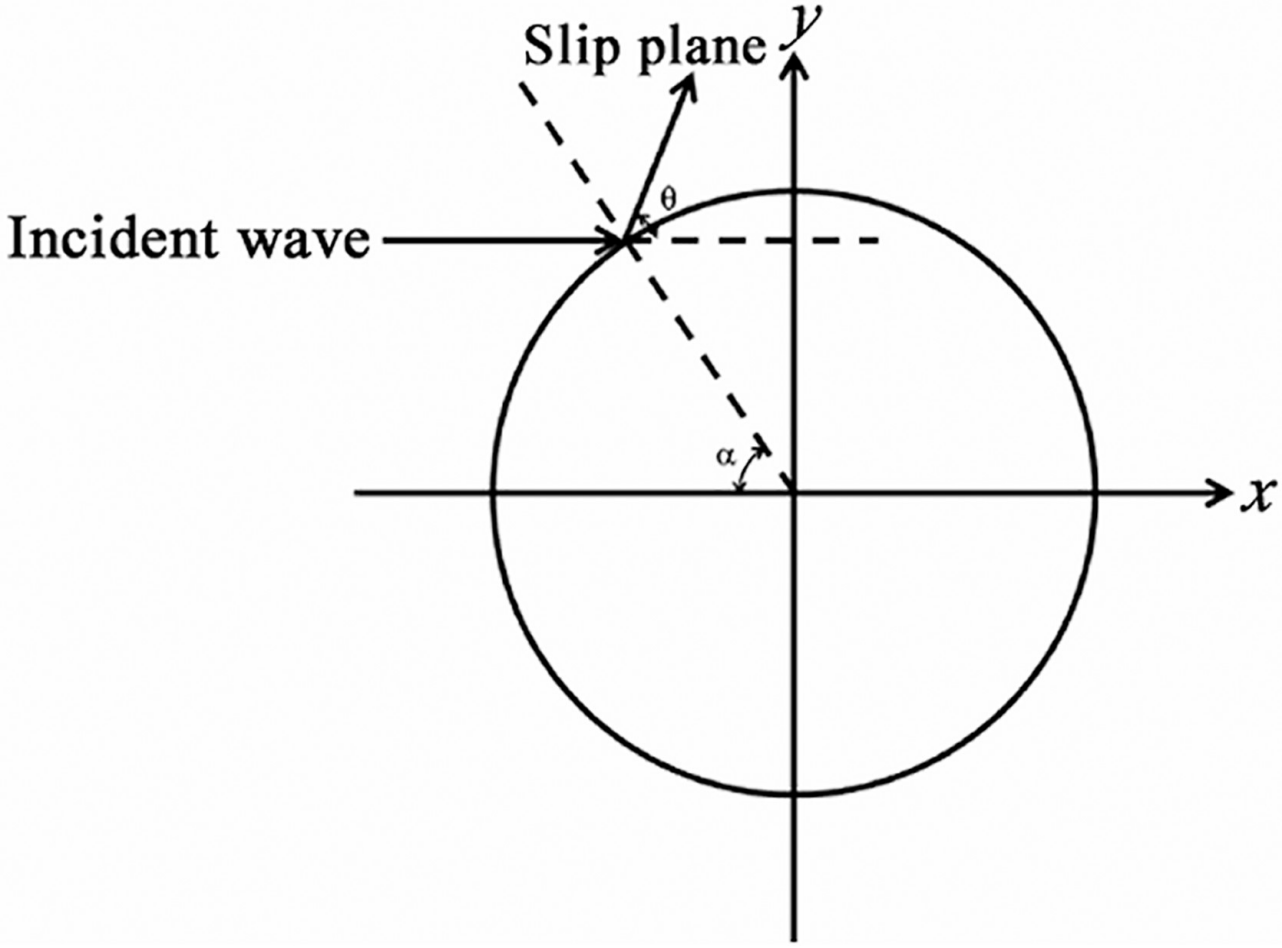
Diagrammatic sketch of the plane wave reflection on a void.

Under shock compression loadings, the stress field around voids can be approximated by a biaxial far-field compression problem [25]. Therefore, the resolved shear stress *τ*_*θ*_ can be expressed by the following equation,

$$\tau_{\theta }=\frac{\text{1}}{\text{2}}T_{z}\times [2(k-1)]\text{cos}2\alpha +(1+k)]\text{sin}[2(\theta -\alpha )],$$

where, *T*_*z*_ is the far-field compression loading, *k* is the biaxial loading ratio and estimated to be *k* = *c*_12_/*c*_11_ = 60.8 GPa/107.3 GPa = 0.567 for Al under uniaxial strain condition [25, 26], α is the incident angle of the incident wave and varies from -90° to 90°, *θ is* the angle between the load direction (*x* axis) and the {111} slip planes, as illustrated in Fig 7. The earliest-appearing dislocations would nucleate at positions where the resolved shear stress reaches a maximum value. Table 2 lists the maximum values of the resolved shear stress (denote by |*τ*_*θ*_*|*_*max*_) in different lattice orientations.

**Table 2:** Maximum value of the resolved shear stress at different lattice orientations.

Impacting lattice orientation	slip plane	*θ*	|*τ*_*θ*_*|*_*max*_
<100>	$\begin{array}{c} \left(111\right)\\ \left(11\bar{1}\right)\\ \left(\bar{1}11\right)\\ \left(1\bar{1}1\right) \end{array}$	35.3° 35.3° 35.3° 35.3°	1.1976|*T*_*z*_| 1.1976|*T*_*z*_| 1.1976|*T*_*z*_| 1.1976|*T*_*z*_|
<111>	$\begin{array}{c} \left(111\right)\\ \left(11\bar{1}\right)\\ \left(\bar{1}11\right)\\ \left(1\bar{1}1\right) \end{array}$	^90°^ ^19.469°^ ^19.469°^ ^19.469°^	0.8769|*T*_*z*_| 1.0953|*T*_*z*_| 1.0953|*T*_*z*_| 1.0953|*T*_*z*_|
<110>	$\begin{array}{c} \left(111\right)\\ \left(11\bar{1}\right)\\ \left(\bar{1}11\right)\\ \left(1\bar{1}1\right) \end{array}$	54.726° 54.726° 0° 0°	1.1964|*T*_*z*_| 1.1964|*T*_*z*_| 0.8769|*T*_*z*_| 0.8769|*T*_*z*_|
<112>	$\begin{array}{c} \left(111\right)\\ \left(11\bar{1}\right)\\ \left(\bar{1}11\right)\\ \left(1\bar{1}1\right) \end{array}$	0° 70.553° 28.12° 28.12°	0.8769|*T*_*z*_| 1.0903|*T*_*z*_| 1.1605|*T*_*z*_| 1.1605|*T*_*z*_|
<130>	$\begin{array}{c} \left(111\right)\\ \left(11\bar{1}\right)\\ \left(\bar{1}11\right)\\ \left(1\bar{1}1\right) \end{array}$	21.417° 21.417° 46.913° 46.913°	1.1127|*T*_*z*_| 1.1127|*T*_*z*_| 1.1400|*T*_*z*_| 1.1400|*T*_*z*_|

The unit for |*τ*_*θ*_*|*_*max*_ is GPa.

The earliest-appearing dislocations would nucleate at positions where the resolved shear stress reaches the maximum value, and for the same lattice orientation, dislocations prefer to nucleate on slip planes with the largest |*τ*_*θ*_*|*_*max*_. Therefore, according to the above calculations, we have the following conclusions, (1) for <100> orientation, the earliest-appearing dislocations would nucleate on all {111} slip planes; (2) for <111> orientation, the earliest-appearing dislocations would nucleate on $\left(11\bar{1}\right)$, $\left(\bar{1}11\right)$ and $\left(1\bar{1}1\right)$ slip planes; (3) for <110> orientation, the earliest-appearing dislocations would nucleate on (111) and $\left(11\bar{1}\right)$ slip planes; (4) for <112> orientation, the earliest-appearing dislocations would nucleate on $\left(\bar{1}11\right)$ and $\left(1\bar{1}1\right)$ slip planes; (5) for <130> orientation, the earliest-appearing dislocations would nucleate on $\left(\bar{1}11\right)$ and $\left(1\bar{1}1\right)$ slip planes.

**Fig 8:**
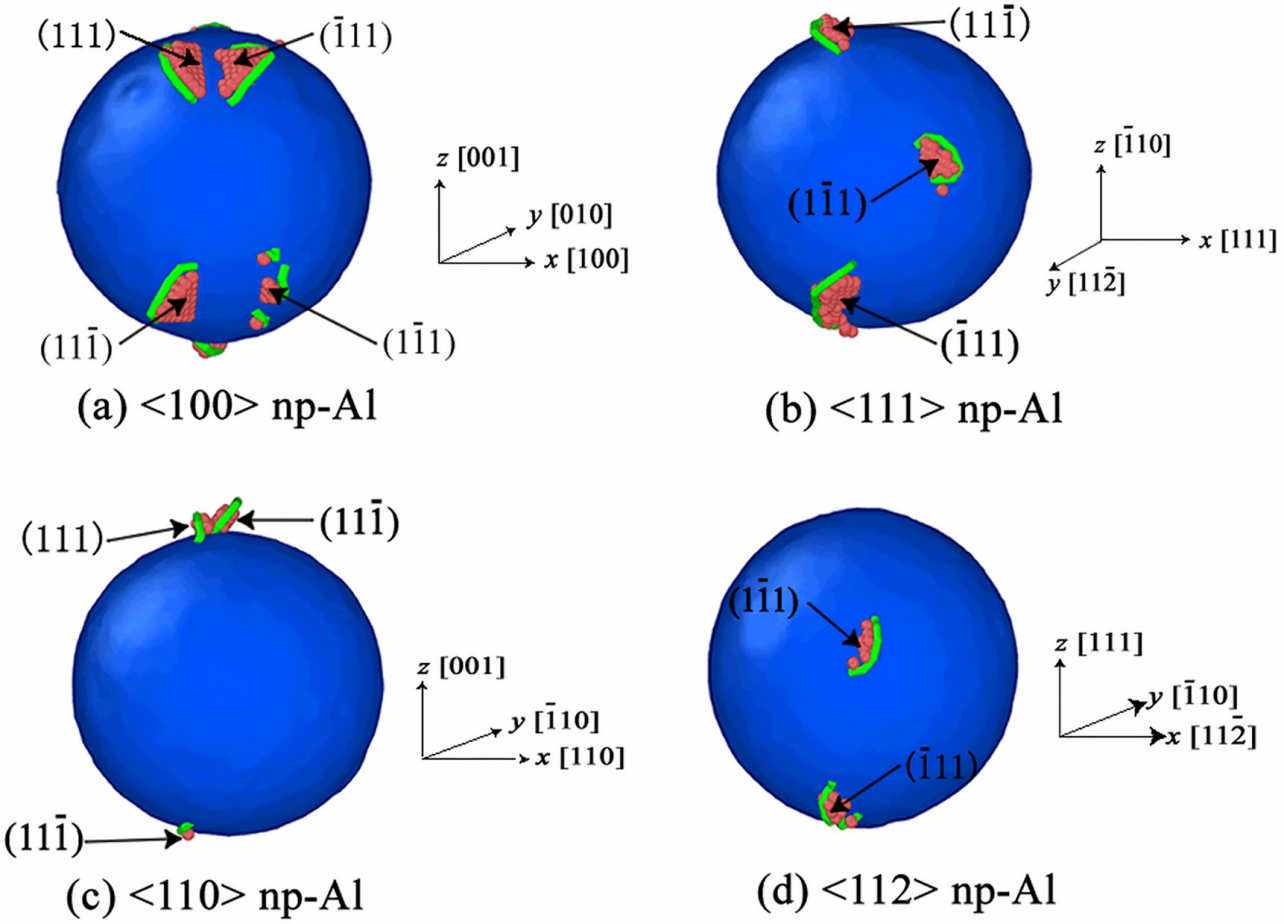
Slip planes where the earliest-appearing dislocations nucleate on at different lattice orientations under *v* = 0.2 km/s.

Fig 8 displays the slip planes where the earliest-appearing dislocations prefer to nucleate on in the case of *v* = 0.2 km/s. Fig 8(a) shows dislocations begin to nucleate on all {111} slip planes for <100> orientation; Fig 8(b) illustrates that dislocations prefer to nucleate on $\left(11\bar{1}\right)$, $\left(\bar{1}11\right)$ and $\left(1\bar{1}1\right)$ slip planes for <111> orientation; Fig 8(c) demonstrates that dislocations nucleate on (111) and $\left(11\bar{1}\right)$ slip planes in priority for <110> orientation, while dislocations would nucleate on $\left(\bar{1}11\right)$ and $\left(1\bar{1}1\right)$ slip planes for <112> orientation, the four simulation results agree well with the theoretical model of the stress distribution on the surface of void; however, in our simulations, no dislocation is detected in <130> np-Al sample under *v* = 0.2 km/s, which seems to be contradicted with the theoretical model of the stress distribution. Actually, **|*τ***_***θ***_***|***_***max***_ is determined by *T*_*z*_, which is the compression stress along the shock direction. The values of *T*_*z*_ at the initial compression stage (eg. *t* = 2.0 ps) are calculated to be -3.43 GPa, -3.47 GPa, -3.52 GPa, -3.37 GPa and -3.25 GPa for <100>, <111>, <110>, <112> and <130> orientations, respectively; and the corresponding **|*τ***_***θ***_***|***_***max***_ is 4.11 GPa, 3.80 GPa, 4.21 GPa, 3.91 GPa and 3.70 GPa. The value of **|*τ***_***θ***_***|***_***max***_ for <130> orientation is found to be the minimum value in all orientations. An reasonable explanation for the absence of dislocation in <130> orientation under *v* = 0.2 km/s is that the resolved shear stress is too small to reach the critical value for dislocation to nucleate.

**Fig 9:**
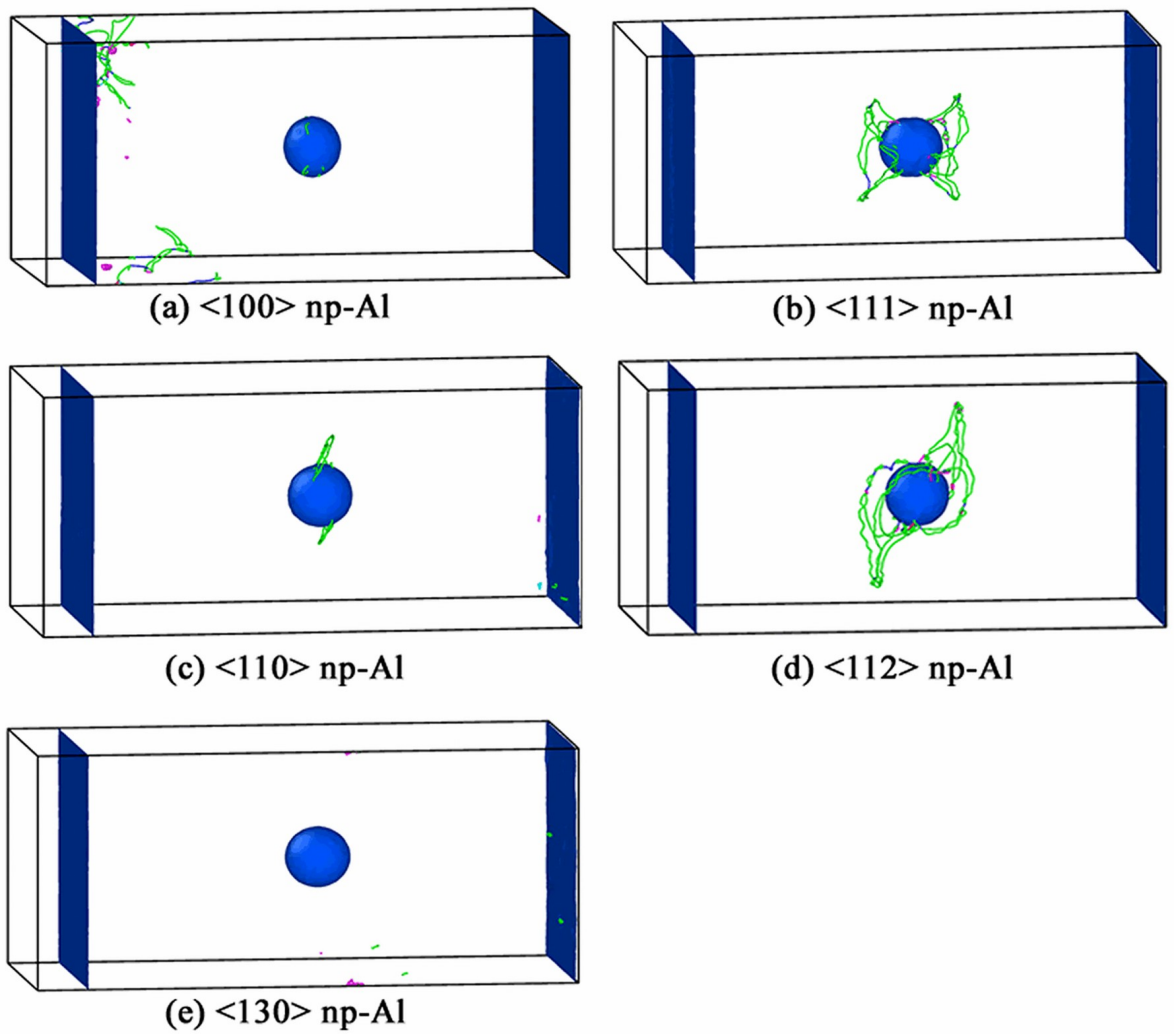
Dislocation activities in all simulated np-Al samples under *v* = 0.2 km/s at *t* = 40 ps.

The dislocation nucleation process in all simulated np-Al samples under *v* = 0.2 km/s at time *t* = 40 ps are displayed in Fig 9. For <100> np-Al sample, dislocations are found to emit from both the loading surface and void surface, as seen in Fig 9(a); for <111>, <110> and <112> np-Al samples, dislocations are observed to emit from void surface rather than from the loading surface, as shown in Fig 9(b)–9(d); however, for <130> np-Al sample, no dislocation is detected during the whole simulation process. Unlike cases in *v* = 0.5 km/s and 1.0 km/s, voids in all simulated np-Al samples are not collapsed under *v* = 0.2 km/s.

**Fig 10:**
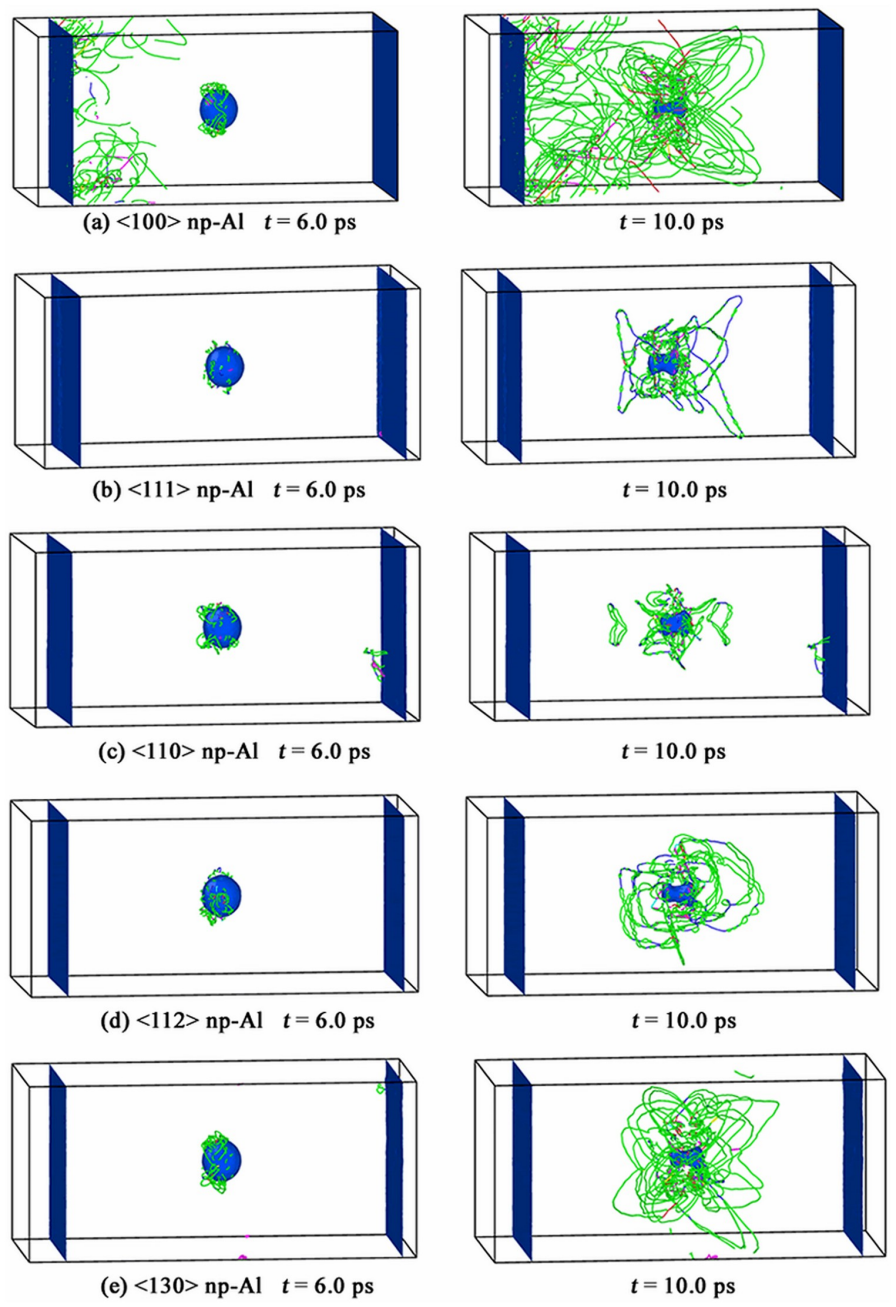
Dislocation activities in all simulated np-Al samples under *v* = 0.5 km/s.

Fig 10 shows the dislocation nucleation process in all simulated np-Al samples under *v* = 0.5 km/s. For <100> np-Al sample, dislocations are found to emit from both the impacting surface and void surface, which is similar to the case of *v* = 0.2 km/s, as seen in Fig 10(a); for <111>, <110>, <112> and <130> np-Al samples, dislocations are emitted from void surface rather than from the impacting surface, as shown in Fig 10(b)–10(e).

**Fig 11:**
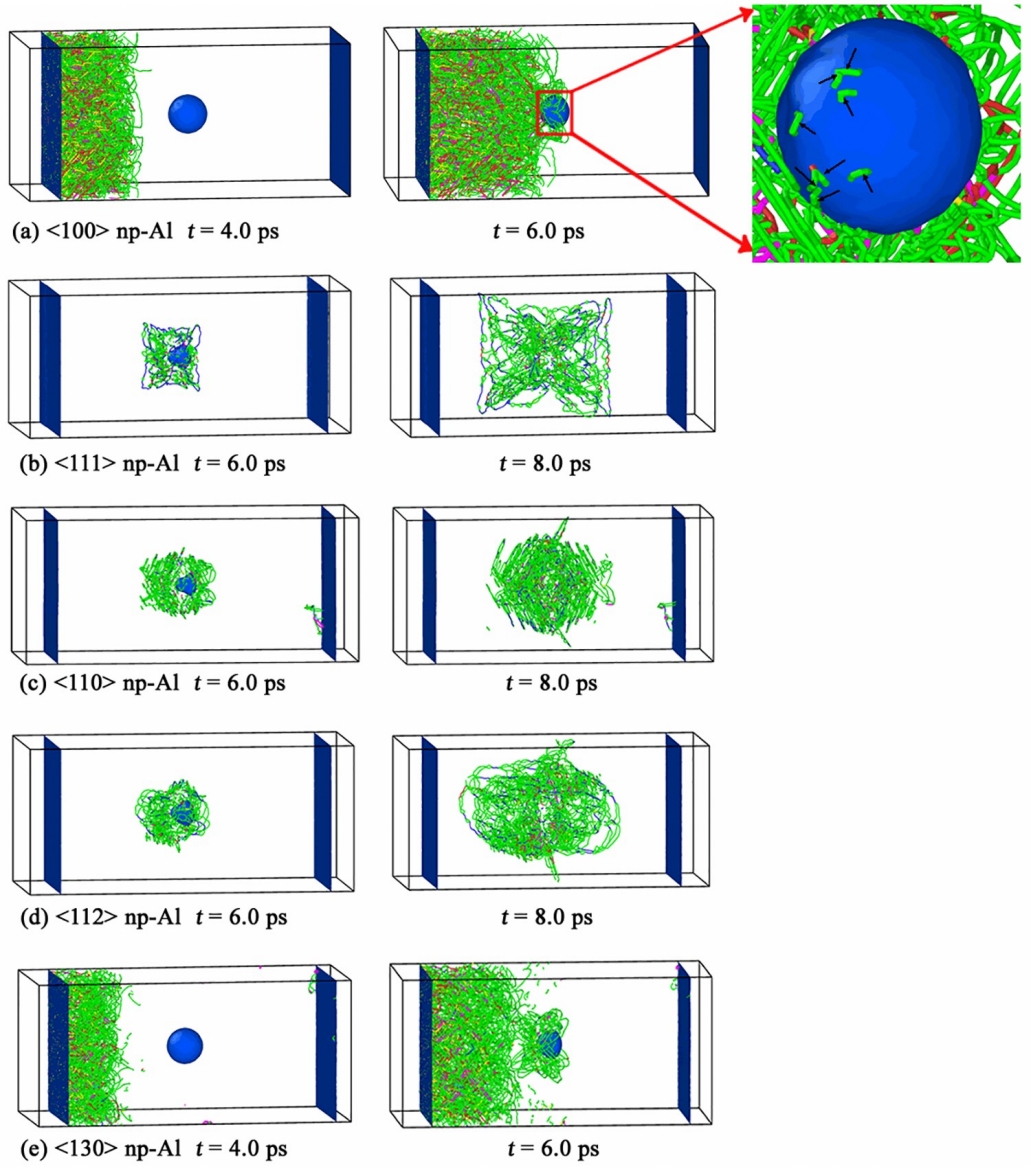
Dislocation activities in all simulated np-Al samples under *v* = 1.0 km/s. The black arrows in the right figure of Fig. (a) indicate dislocations emitted from the surface of the void.

When the piston velocity increases to *v* = 1.0 km/s, homogeneous dislocation nucleation occurs in the void-free bulk zone before the shock wave reaches the void surface in <100> np-Al sample, as shown in the left figure of Fig 11(a). With further deformation, more and more dislocations are emitted from the impacting surface and gliding on the slip planes. When the elastic precursor reaches the void surface at *t* = 6.0 ps, dislocations begin to nucleate from the surface of void, as seen in the right figure of Fig 11(a), where, the black arrows indicate dislocations emitted from the surface of the void. Fig 11(b)–11(d) illustrate that dislocations are emitted from the surface of void in <111>, <110> and <112> np-Al samples under *v* = 1.0 km/s, which is similar to cases of *v* = 0.2 km/s and 0.5 m/s. For <130> np-Al sample, dislocations are emitted from the loading surface before the shock wave reaches the void surface. Once the shock wave gets to the void surface, dislocations nucleate from the surface of void immediately, as seen in Fig 11(e).

## Conclusions

Anisotropic shock responses of nanoporous Al under shock compression are systematically investigated by non-equilibrium molecular dynamics simulations. A transverse collapse of voids is found to happen under a weaker shock, while a longitudinal collapse of voids occurs under a stronger shock. Due to the plastic behaviors around the void, the von Mises stress nearby the void exhibited similar distribution to that of the von Mises strain. The shape evolution of void during collapse is independent of the impacting crystallographic orientation. The dislocation nucleation position is strongly depended on the impacting crystallographic orientation. A theory based on resolved shear stress is used to explain differences in dislocation behaviors under different shock orientations. The results obtained in the present work can serve as important references for constructing micromechanism- based continuum models for void collapse at meso-to-macro scales.

## Supporting information

S1 File (DOC)
