# Supplementary material for: Anisotropic shock responses of nanoporous Al by molecular dynamics simulations
Source: PLoS One. 2021 Mar 17;16(3):e0247172. doi: 10.1371/journal.pone.0247172 (PMC7968703; doi:10.1371/journal.pone.0247172)
Supplement: S1 File — (DOC) [file pone.0247172.s001.doc]

In our manuscript, only the data of the lattice constant of Al (0.4049 nm) is used.
